# RETRACTED: Quazi et al. The Discovery of Novel Antimicrobial Agents Through the Application of Isocyanide-Based Multicomponent Reactions. *Antibiotics* 2023, *12*, 849

**DOI:** 10.3390/antibiotics14090921

**Published:** 2025-09-12

**Authors:** Sameer Quazi, Maliha Tabassum Rashid, Javid Ahmad Malik, Shreelaxmi Gavas

**Affiliations:** 1 GenLab Biosolutions Private Limited, Bangalore 560043, Karnataka, India; 2 Department of Biomedical Sciences, School of Life Sciences, Anglia Ruskin University, Cambridge CB1 1PT, UK; 3 School of Health Sciences, Faculty of Biology, Medicine and Health, The University of Manchester, Manchester M13 9PL, UK; 4 SCAMT Institute, ITMO University, St. Petersburg 197101, Russia; 5 Asian Network of Research on Anti-Diabetic Plants (ANRAP), Dhaka 1216, Bangladesh; rashidmalihatabassum@gmail.com; 6 Department of Zoology, Guru Ghasidas University, Bilaspur 495009, Chhattisgarh, India; jvdmalik8@gmail.com; 7 Navya Care, Bangalore 560001, Karnataka, India; shreelaxmi012@gmail.com

The *Antibiotics* Editorial Office retracts the paper “The Discovery of Novel Antimicrobial Agents through the Application of Isocyanide-Based Multicomponent Reactions” [1], cited above.

Following publication, concerns were brought to the attention of the publisher regarding the authenticity of the authorship of this article [1].

Adhering to our complaint procedure, an investigation was conducted by the Editorial Office and Editorial Board. Following communications with the authorship group, the Editorial Board were unable to verify the identity, contribution, or affiliations of a number of the authors listed on this manuscript, nor could the origins of the study be confirmed. As a consequence, the Editorial Board has lost confidence in the integrity of the findings and has decided to retract this publication [1] as per MDPI’s retraction policy (https://www.mdpi.com/ethics#_bookmark30).

This retraction was approved by the Editor-in-Chief of the journal *Antibiotics*.

The corresponding author, Sameer Quazi, agreed to the retraction. Maliha Tabassum Rashid and Javid Ahmad Malik wish to indicate that they were not aware of this submission nor consented to its publication. Shreelaxmi Gavas did not provide a comment on this decision.
